# Restoration of FBP1 suppressed Snail-induced epithelial to mesenchymal transition in hepatocellular carcinoma

**DOI:** 10.1038/s41419-018-1165-x

**Published:** 2018-11-14

**Authors:** Gao-Min Liu, Qiao Li, Peng-Fei Zhang, Shun-Li Shen, Wen-Xuan Xie, Bin Chen, Jian Wu, Wen-Jie Hu, Xiao-Yong Huang, Bao-Gang Peng

**Affiliations:** 1 Department of Liver Surgery, The First Affiliated Hospital, Sun Yat-sen University, No. 58 Zhongshan Er Road, 510080 Guangzhou, China; 2 Department of Hepatobiliary Surgery, Meizhou People’s Hospital, No. 34 Huangtang Road, 514031 Meizhou, China; 3 Liver Cancer Institute, Zhongshan Hospital, Fudan University, Shanghai, China

## Abstract

Fructose-1,6-bisphosphatase (FBP1), one of the rate-limiting gluconeogenic enzymes, plays critical roles in several cancers and is treated as a tumour suppressor. However, its role in hepatocellular carcinoma (HCC) is unclear. Here, we demonstrated that FBP1 was significantly inhibited during Snail-induced epithelial to mesenchymal transition (EMT) and tissues in HCC. Restoration of FBP1 expression in HCC cancer cells suppressed EMT phenotype, tumour migration and tumour growth induced by Snail overexpression in SMMC-7721 cells. Gene set enrichment analyses revealed significantly enriched terms, including WNT, Notch, ESC, CSR and PDGF, in the group with high Snail and low FBP1 compared with those with low Snail and high FBP1. Low FBP1 expression was significantly correlated with higher AFP level, satellite nodules, portal vein tumour thrombus, and advanced tumour stage. Survival analyses showed that FBP1 was an independent prognostic factor for overall survival and recurrence-free survival. In conclusion, our study revealed a vital role for FBP1 in Snail-induced EMT and prognostic prediction in HCC.

## Introduction

Hepatocellular carcinoma (HCC) remains a global public health issue. It is ranked as the fifth leading cancer and the second leading cause of cancer-related mortality^1^. The long-term survival is far from satisfying due to the low rate of curative treatment and the high rate of post-curative recurrence. There is always an urgent demand for a better understanding of the molecular mechanisms underlying HCC^2^.

The ability of differentiated epithelial cells to acquire mesenchymal traits during embryonic development, wound healing, malignant tumour progression and chemoresistance is now termed epithelial–mesenchymal transition (EMT). Via EMT, cells acquire mesenchymal properties, such as expression of Vimentin, but at the same time lose the expression of epithelial markers, such as E-cadherin^3^. Snail is one of the most important transcription factors that drive EMT^4,5^. However, little is known about the role of aberrant glucose metabolism in Snail-induced EMT.

Glucose homeostasis is reciprocally controlled by the catabolic glycolysis/oxidative phosphorylation (OXPHOS) and the anabolic gluconeogenesis pathway. Aberrant glucose metabolism promotes tumourigenesis and progression in many cancers^6^. As first described in 1920s, some tumour cells preferentially rely on glycolysis rather than OXPHOS, even in conditions with ample oxygen (“aerobic glycolysis” or “Warburg effect”)^7^. While previous studies have paid much attention to abnormal glycolysis, little effort has been made to understand the role of gluconeogenesis, the reciprocal metabolic process of glycolysis, in cancer. Fructose-1,6-bisphosphatase (FBP1), one of the rate-limiting enzymes in gluconeogenesis, catalyses the hydrolysis of fructose-1,6-bisphosphate (F-1,6-P2) to fructose 6-phosphate (F-6-P) and inorganic phosphate. Recently, FBP1 was reported to play suppressive–suppressive roles in several cancers, including renal cancer^8^, breast cancer^9^, lung cancer^10^, pancreatic cancer^11,12^ and gastric cancer^13^. Until now, the most important mechanism underlying FBP1 suppression was reported to be promoter DNA methylation^9,10,14,15^. However, the role and mechanism of dysregulated FBP1 in HCC remain far from clear. We conducted this study to clarify the role of FBP1 in Snail-induced EMT and the prognostic role of FBP1 in HCC.

## Materials and methods

### Clinical specimens, tissue microarray and immunohistochemistry

All patients were enrolled from the First Affiliated Hospital of Sun Yat-sen University between January 2006 and December 2009. The median follow-up for the 242 patients was 31.0 months (range, 3–95 months). Patients were staged according to the seventh edition of the International Union Against Cancer TNM classification system. All protocols were approved by the Ethics and Indications Committee of the First Affiliated Hospital of Sun Yat-sen University. Written informed consent was obtained from all patients.

A tissue microarray (TMA) of 242 pairs of HCCs and corresponding peritumoural tissues was constructed. The tissue samples were incubated with anti-Snail (Abcam, #ab180714, 1:100), anti-FBP1 (Abcam, #ab180714, 1:200) and E-cadherin (Cell Signaling Technology, #9782, 1:400) antibodies overnight at 4 °C. Negative controls were performed without primary antibodies. Three representative fields were imaged in a uniform setting for all slides. Image-Pro Plus v6.2 software (Media Cybernetics Inc., Bethesda, MD) was used to measure the density of positive staining. The median values were defined as the cutoff values for high and low Snail or FBP1 expression.

### Cell culture

Seven human liver cancer cell lines (Bel-7402, Hep3B, HepG2, MHCC-97H, MHCC-97L, PLC/PRF/5 and SMMC-7721) were used for the analyses. All cell lines were obtained from the Chinese Academy of Sciences. Unless specifically indicated, cells were cultured in Dulbecco’s modified Eagle’s medium (DMEM) containing 10% foetal bovine serum (Gibco, USA) at 37 °C with 5% CO_2_ and 95% humidity. Quantitative real-time PCR (qRT-PCR) total RNA was extracted with a TRIzol (Gibco, USA) protocol and reverse transcribed into complementary DNA (cDNA) using the RevertAid TM First Strand cDNA Synthesis kit (Thermo, USA). qRT-PCR was performed using the BestarSybrGreen qPCR master mix kit (DBI, Germany) and BIO-RAD IQ5qRT-PCR System (Bio-Rad, USA). Next, mRNA levels were normalised against glyceraldehyde-3-phosphate dehydrogenase (GAPDH) and β-tubulin. The following primers were used: human Snail, forward 5ʹ-CAATCGGAAGCCTAACTACAGC-3ʹ and reverse 5ʹ-GACAGAGTCCCAGATGAGCA-3ʹ; human E-cadherin, forward 5ʹ-GGGTTATTCCTCCCATCAGC-3ʹ and reverse 5ʹ-GTCACCTTCAGCCATCCTGT-3ʹ; human Vimentin, forward 5ʹ-AAGAGAACTTTGCCGTTGAAG-3ʹ and reverse 5ʹ-ACGAAGGTGACGAGCCATT-3ʹ; human FBP1, forward 5ʹ-GATTGCCTTGTGTCCGTTG-3ʹ and reverse 5ʹ-TGCCATACAGTGCGTAGCC-3ʹ; human GAPDH, forward 5ʹ-GAGTCAACGGATTTGGTCGT-3ʹ and reverse 5ʹ-TTGATTTTGGAGGGATCTCG-3ʹ. The 2^-△CT^ method was used for quantification.

### Western blotting analyses

Cultured cell lysates were prepared using RIPA buffer and Phenylmethanesulfonyl fluoride (PMSF) (both from Beyotime, Nantong, China) and then separated on 10% Sodium dodecyl sulfate (SDS) polyacrylamide gels. Primary antibodies against Snail (Abcam, #ab180714), FBP1 (Abcam, #ab180714), E-cadherin (Cell Signaling Technology, #9782), Vimentin (Cell Signaling Technology, #9782), Slug (Cell Signaling Technology, #9782), zinc-finger E-box-binding homeobox 1 (ZEB1; Abcam, #ab155249) and Twist1 (Abcam, #ab187008) were used. Protein levels were normalised against β-tubulin.

### Cells transfection

The lentiviral-mediated GV320-FBP1 and Penti 5.0-CMV-Snail plasmids were constructed (Shanghai Genechem Co. Ltd). All stably transfected clones were confirmed by qRT-PCR and immunoblotting.

### Transwell migration assay

A total of 5 × 10^4^ cells/well in serum-free DMEM were seeded into the upper chamber of an 8-μm transwell chamber (Beckton Dickinson, Franklin Lakes, NJ, USA); DMEM with 10% bovine serum albumin (BSA) was added in the lower chamber. After 24-h incubation at 37 °C, the cells in the upper chamber were fixed in methanol and then stained with Giemsa solution (Beyotime, Nantong, China). Then, the migrated cells were imaged and quantified.

### CCK8 assay

A total of 1 × 10^5^ cells/well were cultured in 96-well plates. The CCK8 reagent was incubated into each well for 3 h at 37 °C and after 24 h, 48 h, 72 h and 96 h, the Cell Counting Kit-8 was used (Dojindo, Japan). The absorbency of cells at 450 nm was measured using the BioTek Epoch automatic enzyme-labelled instrument (Biotek, USA).

### Immunofluorescence assay

Cells were cultured in six-well plates. After incubation for 48 h, cells were washed with phosphate-buffered saline, fixed with 4% paraformaldehyde, blocked with 5% BSA, stained with E-cadherin and Vimentin, and then stained with Fluorescein isothiocyanate (FITC)-labelled secondary antibody and 4',6-diamidino-2-phenylindole (DAPI) as per the manufacturer’s instructions (Beyotime, Nantong, China). The images were captured using fluorescence microscope.

### Orthotopic transplantation HCC mouse models

Male BALB/c nude mice, aged 4–6 weeks, were used. A total of 2 × 10^6^ tumour cells were injected into subcutaneous regions of nude mice. Subcutaneous tumour tissues with a longitudinal diameter of 1 cm were harvested and were cut into approximately 1 mm^3^ pieces. Then, tumour pieces were transplanted into the left hepatic lobe of the mice. All mice were sacrificed 6 weeks later and analysed. All mice were obtained from Shanghai Institute of Material Medicine and maintained in a pathogen-free environment. Animal care and experimental protocols were in accordance with the guidelines established by Shanghai Medical Experimental Animal Care Commission and were in accordance with regulations for the Administration of Affairs Concerning Experimental Animals and National Institutes of Health Guidelines.

### Public clinical dataset analyses and gene set enrichment analyses (GSEA)

Gene expression and survival profiles, if available, were obtained from the National Cancer for Biotechnology Information Gene Expression Omnibus (GEO) database (http://www.ncbi.nlm.nih.gov/gds, RRID: SCR_005012) (accession number GSE14520, GSE54236, GSE25097)^16–18^. Moreover, mRNA expression and clinical data from 377 LIHC and 50 normal control samples were obtained from The Cancer Genome Atlas (TCGA, last download date: 2017.11.01) (http://cancergenome.nih.gov/, RRID: SCR_003193) and cBioportal for Cancer Genomics (last download date: 2017.11.01) (http://www.cbioportal.org/, RRID: SCR_014555)^19,20^. The exclusion criteria for clinical analyses were: (1) clinical information not confirmed, and (2) not available (NA) or −infinite (−Inf) gene expression values.

GSEA v3.0 (http://www.broadinstitute.org/gsea/, RRID: SCR_003199)^21,22^ was used to find terms predicted to be enriched in C2 (the Kyoto Encyclopaedia of Genes and Genomes pathway, KEGG), in C5 (a gene set that contained genes annotated by the same Gene Ontology (GO) term), and in C6 (oncogenic signatures of gene sets that represent the signatures of cellular pathways that are often dysregulated in cancer). Data from non-HCC patients were excluded. *P* < 0.05 and false discovery rate (FDR) *q*-value < 0.25 were considered statistically significant.

### Statistical analysis

Statistical analyses were performed using SPSS V19.0 (SPSS Inc., USA), R software V3.5.1 (R Foundation for Statistical Computing, Vienna, Austria) and presented using GraphPad Prism v7.00 (GraphPad Software Inc., USA). Qualitative variables were analysed using the Pearson *χ*^2^ test or Fisher’s exact test. Quantitative variables were compared using *t-*test, Pearson’s correlation test or Spearman rank correlation analysis as appropriate. The survival analyses were plotted using the Kaplan–Meier method and were compared using the log-rank test. Multivariate analyses were performed using the Cox regression model method with forward stepwise procedure. *P* < 0.05 was considered statistically significant.

## Results

### FBP1 was suppressed during Snail-induced EMT

**Fig. 1:**
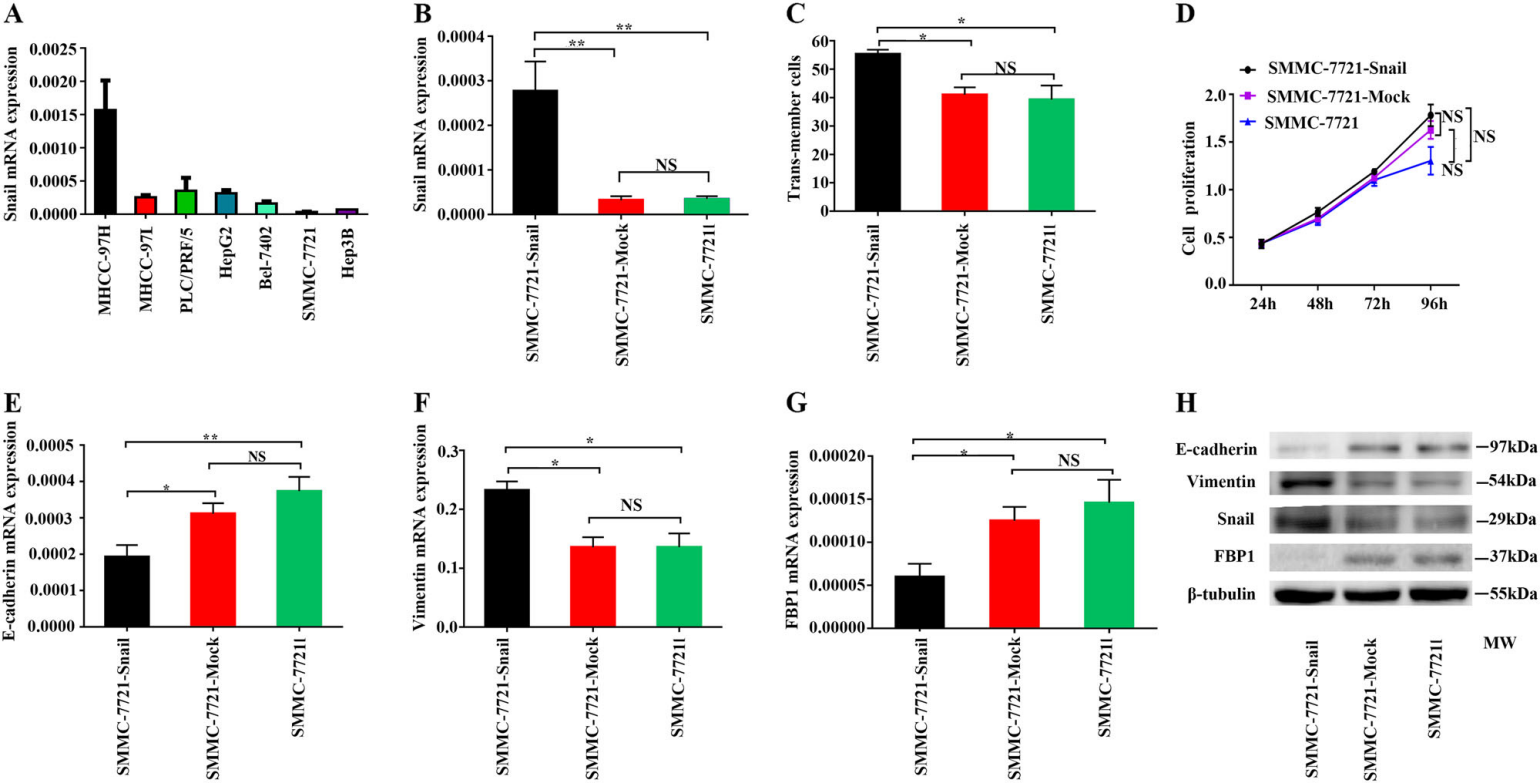
Expression of FBP1 was suppressed during Snail-induced EMT. **a** The expression of Snail in HCC cell lines; Snail was highest in MHCC-97H but lowest in SMMC-7721. **b** Quantitative real-time PCR analysis showed significantly higher expression of Snail in SMMC-7721-Snail than control SMMC-7721 cells. **c** Transwell migration assay showed Snail overexpression significantly promoted SMMC-7721 cell migration. **d** CCK8 assay showed Snail overexpression did not significantly affect SMMC-7721 cell proliferation. **e** Quantitative real-time PCR analysis showed Snail overexpression significantly suppressed E-cadherin in SMMC-7721 cells. **f** Quantitative real-time PCR analysis showed Snail overexpression significantly induced Vimentin in SMMC-7721 cells. **g** Quantitative real-time PCR analysis showed Snail overexpression significantly inhibited FBP1 in SMMC-7721 cells. **h** Western blot analysis showed Snail overexpression induced Vimentin but suppressed E-cadherin and FBP1 protein in SMMC-7721. *P < 0.05, **P < 0.01, NS not significant, compared with control. MW molecular weight. All data are based on three independent repeats

We first examined the expression of Snail using qRT-PCR in seven HCC cell lines: MHCC-97H, MHCC-97L, PLC/PRF/5, HepG2, Bel-7402, SMMC-7721 and Hep3B. SMMC-7721 cells expressed the lowest Snail level, whereas MHCC-97H cells expressed the highest (Fig. 1a). The SMMC-7721 cell line was selected for further experiments. We transfected SMMC-7721 cells with a Snail overexpression plasmid to enhance the expression of Snail (SMMC-7721-Snail cells) (Fig. 1b, h). Upregulation of Snail significantly enhanced cell migration (Fig. 1c) but not cell proliferation (Fig. 1d). Snail overexpression induced Vimentin but suppressed E-cadherin, indicating the induction of EMT (Fig. 1e, f, h).

Next, we tested FBP1 following Snail overexpression. As shown in Fig. 1g, h, Snail upregulation significantly inhibited FBP1 in SMMC-7721 cells. Taken together, these data show that overexpression of Snail expression promoted migration, induced EMT and suppressed FBP1 in HCC. Similar results were found in the Hep3B cell line (Supplemental Fig. 1).

### Expression of FBP1 and EMT markers in multiple HCC cell lines

**Fig. 2:**
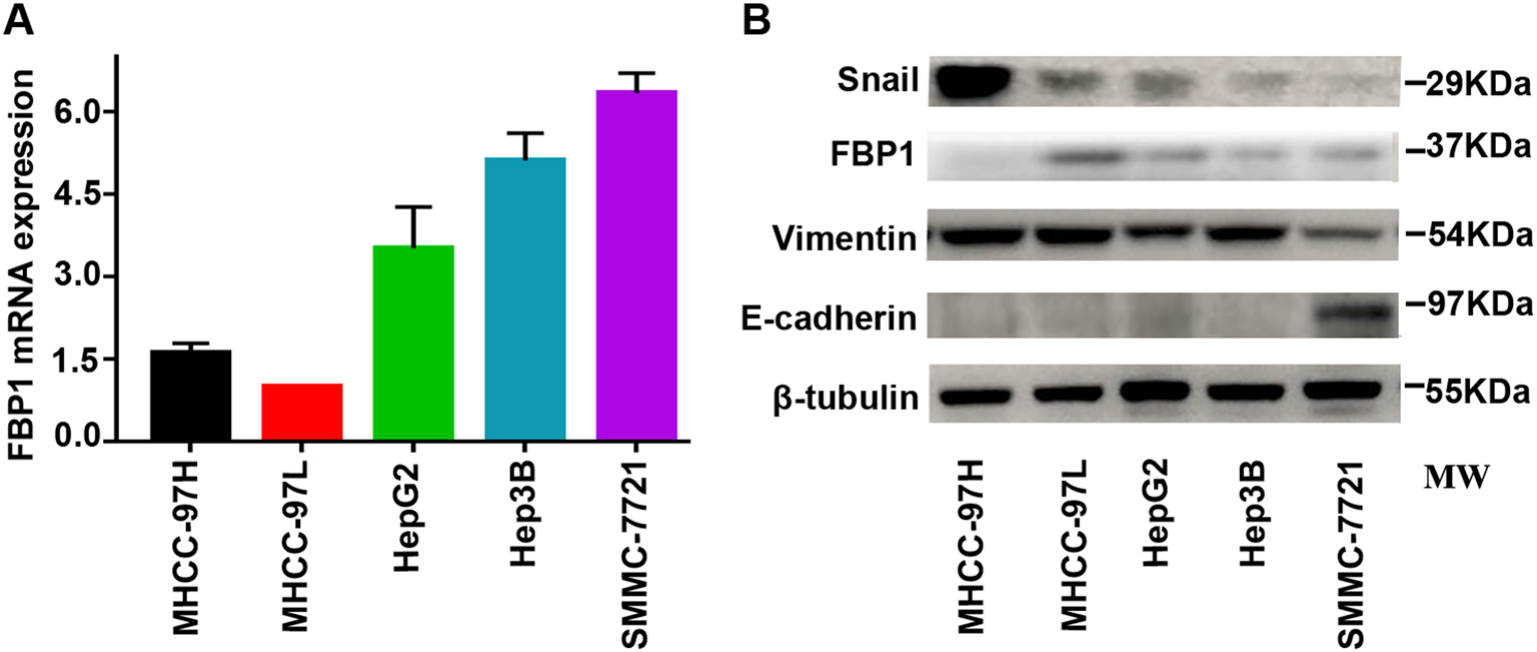
Expression of FBP1 and EMT markers in five HCC cell lines. **a** Quantitative real-time PCR analysis of FBP1 in MHCC-97H, MHCC-97L, HepG2, Hep3B and SMMC-7721 cells. **b** Western blot analysis of FBP1, E-cadherin, Vimentin and Snail in MHCC-97H, MHCC-97L, HepG2, Hep3B and SMMC-7721 cells. All data are based on three independent repeats. MW molecular weight

We analysed FBP1 expression by qRT-PCR and western blot in the five HCC cell lines MHCC-97H, MHCC-97L, HepG2, Hep3B and SMMC-7721. We observed that MHCC-97H cells with the highest Snail and Vimentin expressed the lowest FBP1 and E-cadherin, but SMMC-7721 cells with the lowest Snail and Vimentin significantly expressed FBP1 and E-cadherin (Fig. 2a, b).

### Ectopic FBP1 expression suppressed Snail-induced EMT and tumour growth in HCC

**Fig. 3:**
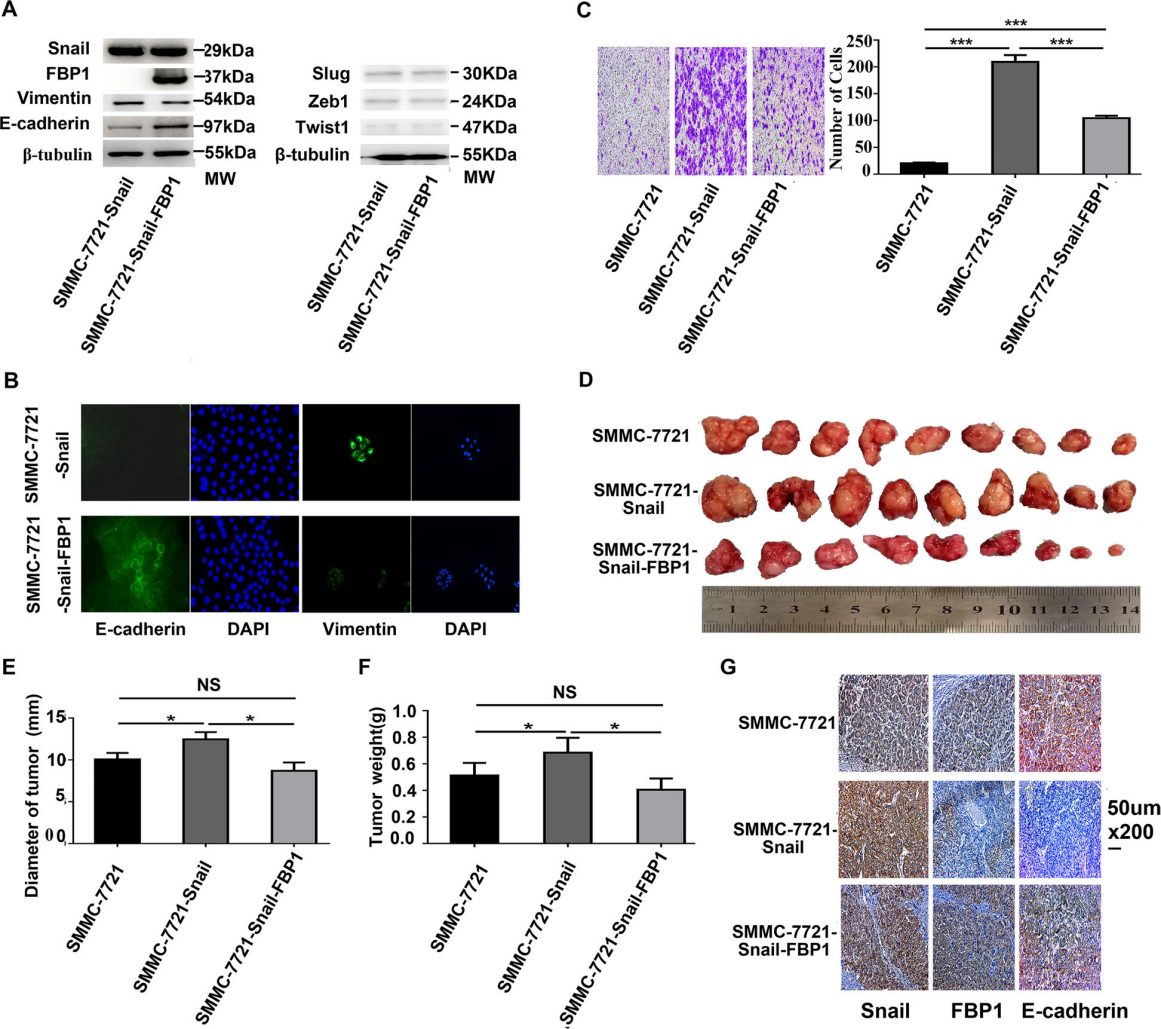
Ectopic FBP1 suppressed Snail-induced EMT and tumour growth in SMMC-7721 cells. **a** Western blot analysis showed expression changes of E-cadherin and Vimentin induced by Snail expression were hindered by forced expression of FBP1 in SMMC-7721 cells. Ectopic FBP1 expression did not significantly affect Snail, Slug, ZEB1 and Twist1 expression. **b** Immunofluorescence assay showed SMMC-7721-snail-FBP1 cells expressed higher E-cadherin but lower Vimentin levels and appeared more like epithelial cells than SMMC-7721-Snail cells. The magnifications used were ×200. **c** Transwell migration analyses showed FBP1 expression significantly inhibited cell migration induced by Snail overexpression in SMMC-7721 cells. **d** Representative images of day 42 tumours in mice transplanted with SMMC-7721, SMMC-7721-Snail and SMMC-7721-Snail-FBP1. **e**, **f** The mean tumour diameters and weights in each group are shown. **g** The representative images of Snail, FBP1 and E-cadherin expression in transplanted tumours. The magnifications used were ×200. *P < 0.05, NS not significant, compared with control. The data presented in **a**–**c** are based on three independent repeats. The number of mice for each group in **d** is 9. MW molecular weight

To further investigate the role of FBP1 in Snail-induced EMT, we upregulated FBP1 expression using the FBP1 overexpression plasmid in SMMC-7721-Snail cells (SMMC-7721-snail-FBP1). SMMC-7721-snail-FBP1 cells expressed higher E-cadherin (Fig. 3a) and appeared more like epithelial cells (Fig. 3b) than SMMC-7721-Snail cells. FBP1 expression significantly inhibited cell migration induced by Snail overexpression (Fig. 3c).

Then, orthotopic transplantation HCC mouse models were established. Overexpression of Snail promoted tumour growth and inhibited E-cadherin (Fig. 3d, e, f). A subcutaneous tumour formation experiment using Hep3B cells revealed similar results (Supplemental Fig. 2). However, ectopic FBP1 significantly inhibited Snail-induced tumour growth (Fig. 3d, e, f) and rescued E-cadherin expression (Fig. 3g). Collectively, these data suggested that FBP1 suppressed Snail-induced EMT, migration and tumour growth in HCC.

### GSEA of TCGA dataset

To further understand the underlying molecular mechanisms of dysregulated FBP1 and Snail, we performed GSEA analyses using data from TCGA LIHC dataset. The expression data of 24,991 genes from 361 HCC patients were included in our analyses. We aimed to identify terms differing between the high (top 10%) and low (bottom 10%) expressing groups of FBP1 and Snail. As FBP1 was suppressed by Snail expression in HCC, we explored the enriched terms overlapping in the high Snail and low FBP1 groups.

**Fig. 4:**
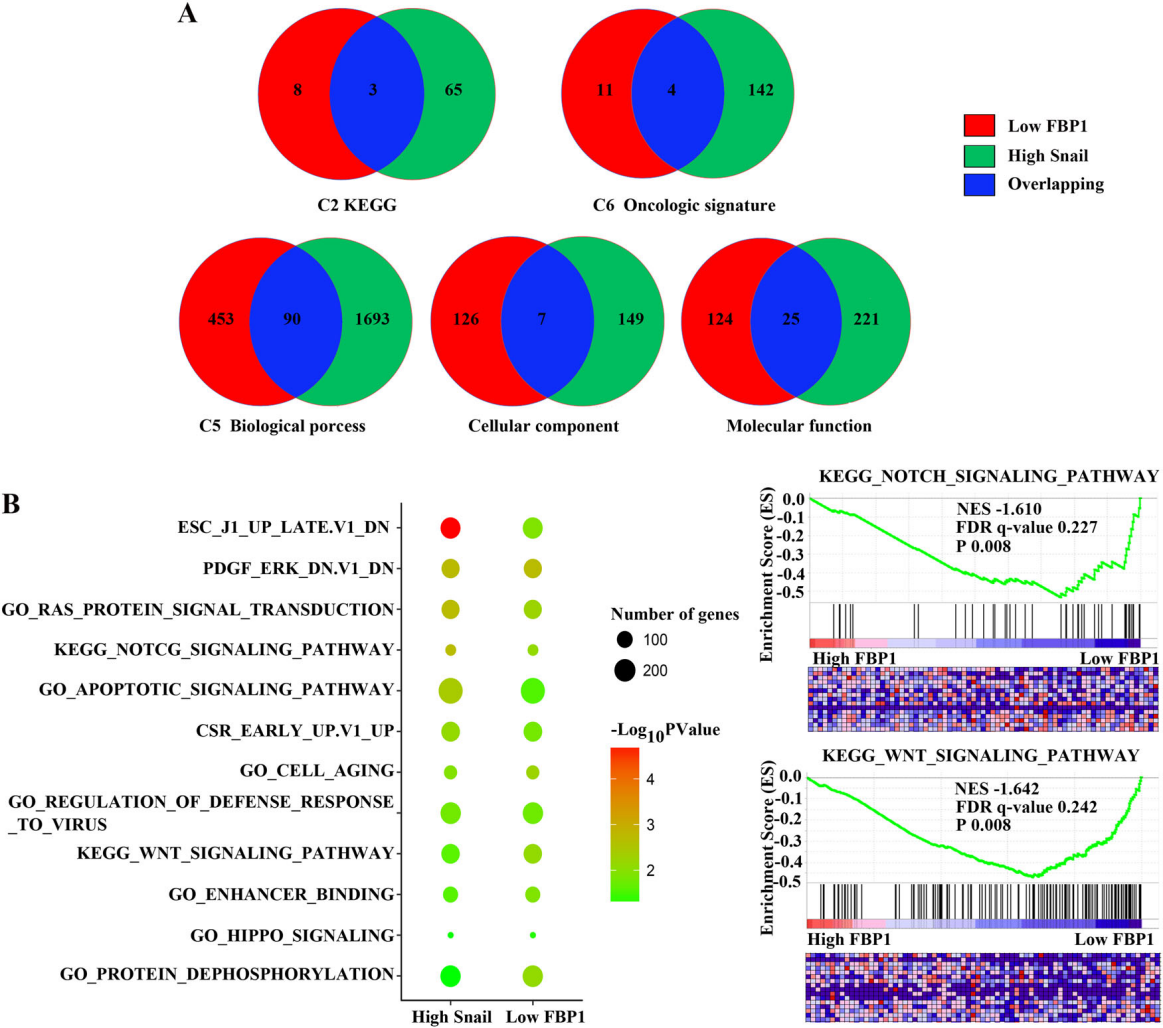
Gene set enrichment analyses using data from TCGA LIHC dataset. **a** The enriched terms in the high Snail group compared with the low Snail group are shown in green. The enriched terms in the low Snail group compared with the high Snail group are shown in red. The overlapping enriched terms are shown in blue. **b** The representative enriched terms are shown. NES normalised enrichment score, FDR false discovery rate

GO terms such as anchoring junction, apoptotic signalling pathway, cell ageing, cytoplasmic region, cell substrate junction, enhancer binding, FC receptor signalling pathway, hippo signalling, positive regulation of cytoplasmic transport, positive regulation of proteolysis, protein dephosphorylation, regulation of defence response to virus, Ras protein signal transduction, KEGG pathways involving WNT signalling pathway, Notch signalling pathway and pathogenic *Escherichia coli* infection were significantly enriched in the high Snail or low FBP1 group when compared with the low Snail or high FBP1 group, respectively. Moreover, oncological signatures including Early serum response (CSR), Dichloroacetate (DCA), Embryonic stem cells (ESC) and Platelet-derived growth factor (PDGF) were found to be significantly associated with high Snail, as well as low FBP1 (Fig. 4, Supplemental Table 1).

### FBP1 was suppressed in HCC patients and indicated prognosis

**Fig. 5:**
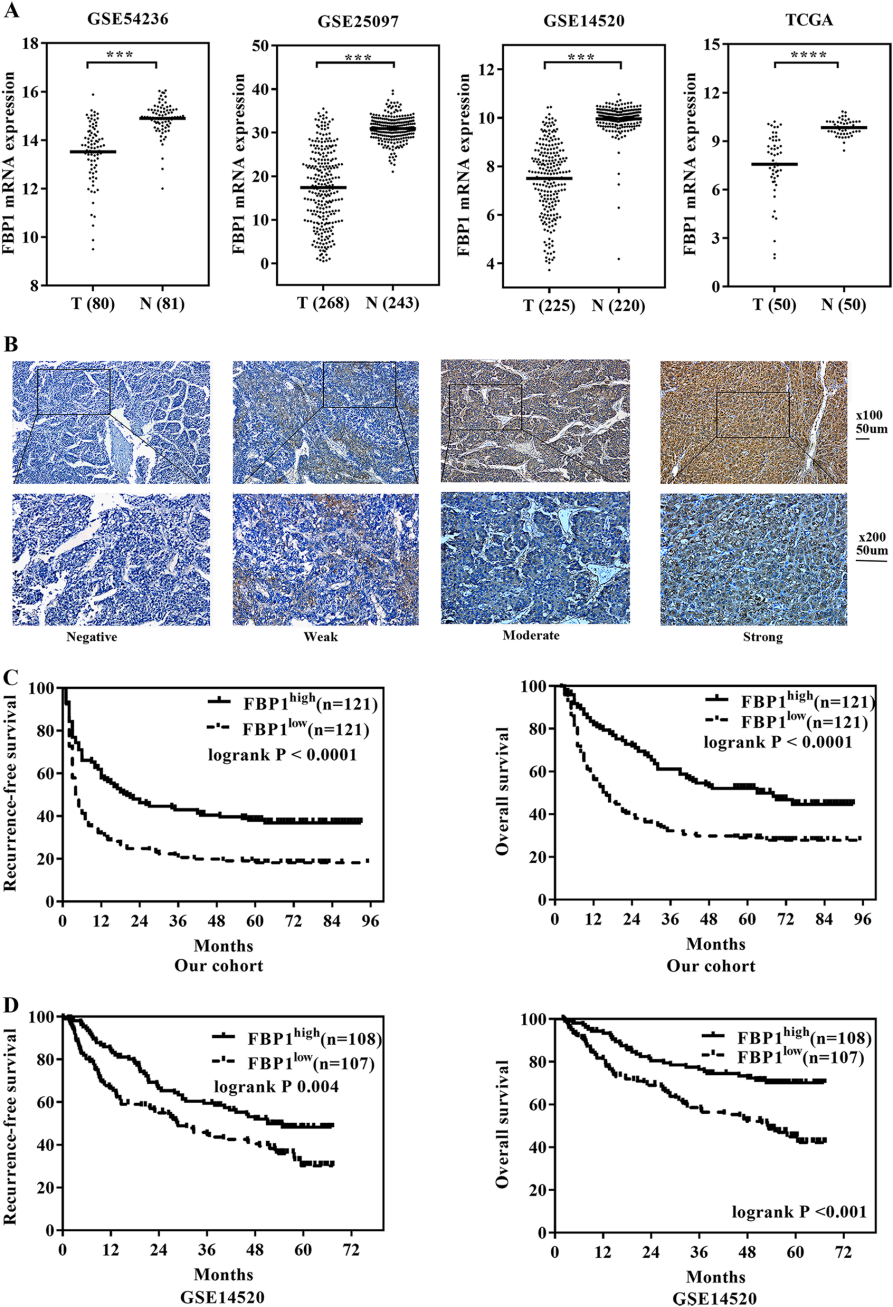
The expression of FBP1 and prognostic role in HCC patients. **a** The expression of FBP1 mRNA was suppressed in HCC when compared with non-tumour controls using data from GSE14520, GSE54236, GSE25097 and TCGA HCC cases (T = tumour tissue, N = non-tumour tissue). **b** The representative images of FBP1 expression in HCC tissues. The magnifications used were ×100 (the upper four) and ×200 (the lower four). **c** Kaplan-Meier survival curves showed significantly better recurrence-free survival and overall survival in patients with high FBP1 expression when compared with those with low FBP1 expression in our cohort. **d** Kaplan–Meier survival curves showed significantly better recurrence-free survival and overall survival in patients with high FBP1 expression when compared with those with low FBP1 expression in the GSE14520 cohort. ***P < 0.001, ****P < 0.0001, when compared with control

We analysed the mRNA expression profile of FBP1 in human HCC using data available in the GEO database, including GSE14520, GSE54236, GSE25097 and TCGA HCC. FBP1 mRNA was found to be significantly suppressed in HCC when compared with non-tumour controls (Fig. 5a).

We then examined the expression of FBP1 in 242 patients from our hospital (Fig. 5b) and classified patients into low and high FBP1 groups using median values as described above. Low FBP1 expression was significantly correlated with alpha-fetoprotein (AFP) ≥ 20 ng/ml (*P* = 0.026), portal vein tumour thrombus (PVTT) (*P* = 0.020), satellite nodule (*P* = 0.006), advanced TNM stage (*P* = 0.001) and advanced Barcelona Clinic Liver Cancer stage (BCLC stage; *P* = 0.001) (Table 1). Kaplan–Meier analyses shown that the 1-, 3-, and 5-year recurrence-free survival (RFS) rates for the high and low FBP1 expression groups were 57.9%, 43.0% and 37.9%, and 31.4%, 20.7% and 18.1%, respectively (*P* < 0.001). The 1-, 3-, and 5-year overall survival (OS) rates for the high and low FBP1 expression groups were 81.8%, 61.2% and 52.0%, and 56.2%, 32.2% and 28.9%, respectively (*P* < 0.001) (Fig. 5c). These were further corroborated by results of the Cox proportional hazard regression analyses, which revealed that FBP1 expression was an independent prognostic factor for RFS and OS (*P* < 0.001 and *P* *=* 0.001, respectively) (Supplemental Table 2, Table 2).

**Table 1:** FBP1 expression and clinicopathological characteristics of 242 HCC patients

Factors	FBP1^low^ (*n* = 121)	FBP1^high^ (*n* = 121)	*P-*value
Sex			
Male	106	107	0.843
Female	15	14	
Age (years)			
≤50	60	61	0.898
>50	61	60	
HbsAg positive			
Yes	110	102	0.119
No	11	19	
HCV positive			
Yes	1	1	1.000
No	120	120	
ALT (IU/L)			
<40	61	64	0.700
≥40	60	57	
Tbil (μmol/L)			
≥17	44	33	0.129
<17	77	88	
ALB (g/L)			
<35	9	5	0.271
≥35	112	116	
AFP (ng/ml)			
<20	23	38	**0.026**
≥20	98	83	
Cirrhosis			
Yes	97	92	0.437
No	24	29	
Tumour diameter			
≤5 cm	36	48	0.105
>5 cm	85	73	
Tumour capsule			
Yes	71	85	0.060
No	50	36	
Satellite nodules			
Yes	48	28	**0.006**
No	73	93	
PVTT			
Yes	29	15	**0.020**
No	92	106	
Edmonson grade			
I+II	93	96	0.641
III+IV	28	25	
TNM stage			
I	60	86	**0.001**
II+III+IV	61	35	
BCLC stage			
A	62	87	**0.001**
B+C	59	34	

*AFP* alpha-fetoprotein, *ALT* alanine aminotransferase, *ALB* albumin, *BCLC stage* Barcelona Clinic Liver Cancer stage, *FBP1* fructose-1,6-bisphosphatase 1, *HBV* hepatitis B virus, *HCV* hepatitis C virus, *TNM stage* tumour-node-metastasis stage, *PVTT* portal vein tumour thrombosis

All the bold P-value were less than 0.05

**Table 2:** Factors associated with RFS and OS significantly found by multivariate analyses in our cohort

Factors	RFS		OS	
	HR (95% CI)	*P*-value	HR (95% CI)	*P*-value
ALB: ≥ 35 vs < 35 g/L	-		0.457 (0.249–0.839)	0.011
Tumour size: > 5 vs ≤ 5 cm	1.997 (1.408–2.832)	<0.001	1.933 (1.313–2.844)	0.001
Encapsulation: complete vs incomplete or absence	0.684 (0.490–0.954)	0.025	0.662 (0.472–0.930)	0.017
Satellite nodules: yes vs no	-		-	
PVTT: yes vs no	1.565 (1.006–2.436)	0.047	-	
Edmonson grade: III+IV vs I+II	-		-	
TNM: II+III+IV vs I	-		-	
BCLC: B+C vs A	1.704 (1.182–2.456)	0.004	2.414 (1.690–3.447)	<0.001
FBP1 expression: high vs low	0.576 (0.424–0.784)	< 0.001	0.582 (0.418–0.809)	0.001

*ALB* albumin, *FBP1* fructose-1,6-bisphosphatase 1, *BCLC stage* Barcelona Clinic Liver Cancer stage, *TNM stage* tumour-node-metastasis stage, *PVTT* portal vein tumour thrombosis

To confirm our results, we then analysed the public dataset GSE14520. In accordance with our cases, low FBP1 expression was correlated with higher AFP level (*P* = 0.001), advanced TNM stage (*P* = 0.022) and advanced BCLC stage (*P* = 0.006). Low FBP1 expression was found to be correlated with larger tumour size (*P* = 0.014) (Supplemental Table 3). Low FBP1 expression was significantly associated with poorer RFS and OS (*P* < 0.001 and *P* = 0.004, respectively) (Fig. 5d). FBP1 expression was also an independent prognostic factor for OS in this cohort (Supplemental Table 4). Collectively, low FBP1 expression indicated poorer prognosis for HCC patients.

### Prognostic role of FBP1 and Snail

**Fig. 6:**
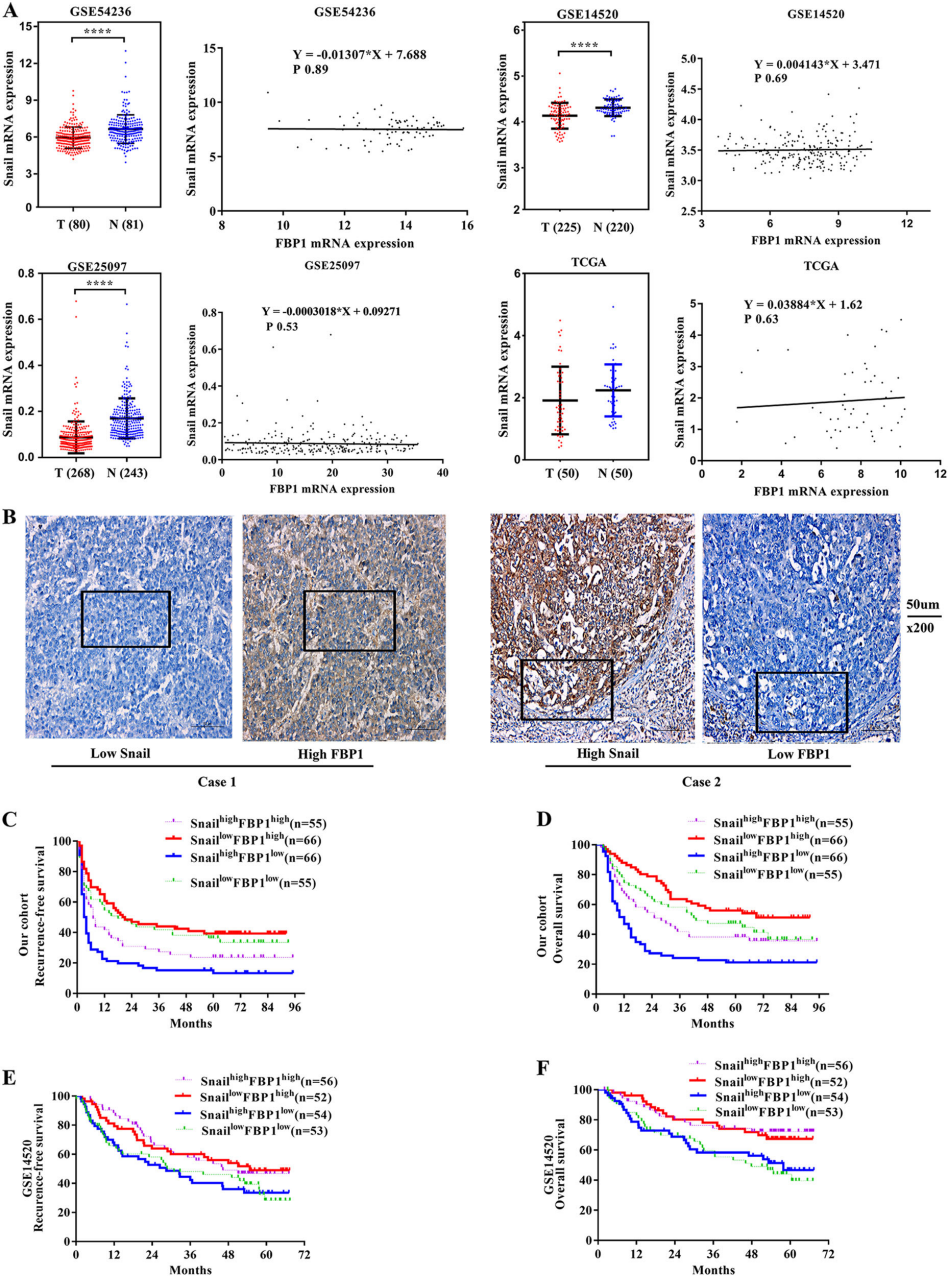
Prognostic role of FBP1 and Snail. **a** The expression of Snail mRNA and its correlation with FBP1 mRNA in GSE14520, GSE54236, GSE25097 and TCGA HCC cases. **b** Representative case with low Snail but high FBP1 expression (Case 1) and case with high Snail but low FBP1 expression (Case 2). The magnifications used were ×200. **c** Kaplan–Meier survival curves showed the difference in recurrence-free survival according to expression of FBP1 and Snail in our cohort. **d** Kaplan–Meier survival curves showed the difference in overall survival according to expression of FBP1 and Snail in our cohort. **e** Kaplan–Meier survival curves showed the difference in recurrence-free survival according to expression of FBP1 and Snail in the GSE14520 cohort. **f** Kaplan–Meier survival curves showed the difference in overall survival according to expression of FBP1 and Snail in the GSE14520 cohort. **** means *P*<0.0001

We investigated the expression of Snail mRNA in GSE14520, GSE54236, GSE25097 and TCGA HCC datasets. As shown in Fig. 6a, Snail mRNA was lower in tumour than non-tumour tissue, and no significant correlation between FBP1 and Snail mRNA was found. We then examined the expression of Snail in our cohort (Fig. 6b). An almost but not quite significant correlation between FBP1 and Snail mRNA was found (*R* = –0.091, Pearson correlation *P* = 0.079). Patients were further divided into four groups: Snail^high^FBP1^high^, Snail^low^FBP1^high^, Snail^high^FBP1^low^ and Snail^low^FBP1^low^. We found that the prognosis of patients in the Snail^low^FBP1^high^ group tended to be the best, whereas the prognosis of patients in the Snail^high^FBP1^low^ tended to be the worst among the four groups (Fig. 6c, d). Similar results were found in the GSE14520 cohort (Fig. 6e, f).

## Discussion

Mounting evidence shows the critical role of aerobic glycolysis in tumourigenesis and progression of cancer^9,13^. In contrast, few studies directly focus on the role of aberrant gluconeogenesis in cancers. Here, we observed suppression of FBP1 during Snail-induced EMT in HCC. We revealed that forced expression of FBP1 repressed the EMT phenotype, HCC migration and tumour growth induced by Snail overexpression. We elucidated several significantly enriched terms, KEGG pathways and oncological signatures in the high Snail and low FBP1 group when compared with the low Snail and high FBP1 group. Last but not least, FBP1 expression was found to be an independent prognostic factor for RFS and OS in HCC.

FBP1 dysregulation provided metabolic advantages and promoted cancer progression in various cancers, including renal cancer^8^, breast cancer^9^, HCC^23,24^, lung cancer^10^, pancreatic cancer^11,12^ and gastric cancer^13^. For example, Li et al. reported that FBP1 hindered renal carcinoma progression by inhibiting Hypoxia-inducible factor (HIF)-1α (HIF1α) activity in the nucleus via a direct interaction in an enzyme-activity-independent manner^8^. FBP1 suppression resulted in an increased cancer stem cell (CSC)-like phenotype and tumourigenesis by enhancing the interaction of β–catenin with T-cell factor in basal-like breast cancer cells^9^. FBP1 overcame gemcitabine resistance and inhibited extracellular regulated protein kinases (ERK) activation by blocking IQ motif containing GTPase-activating protein 1 (IQGAP1)–mitogen-activated protein kinase (MAPK) interaction in pancreatic cancer cells^12^. Low FBP1 expression was found to be an independent factor for poorer survival, which were consistent with previous studies^25^, as well as analyses from public datasets^26,24^ in HCC. From this point of view, FBP1 could serve as suppressive factor in cancer. In contrast, Chen et al. reported that FBP1 expression improved viability and deteriorated survival of breast cancer brain metastatic cells, indicating an oncogenic role for FBP1^27^. All these results implicate that FBP1 exerts different roles in different tissues and stages of cancer.

Promoter DNA methylation is the best understood mechanism for FBP1 loss in cancer. The Rat Sarcoma (RAS)/NF-κB: nuclear factor-kappa B (NF-κB) pathway promoted DNA methylation of FBP1 in gastric cancer cells^14^. The Snail-G9a-Dnmt1 complex, which is critical for E-cadherin promoter silencing, was also responsible for the promoter methylation of FBP1 in basal-like breast cancer cells^9^. ZEB1 interacted with the FBP1 promoter to enhance DNA methylation in lung cancer cells^10^. NPM1 bound directly to the FBP1 promoter region to suppress the expression of FBP1 in pancreatic cancer cells^11^. In addition, copy number loss^8,24^ and post-translational ubiquitin-mediated degradation^28^ were also found to be associated with FBP1 loss. Thus, a complicated and multi-layer regulatory network exists for the suppression of FBP1 in cancer, which remains to be clarified.

EMT promotes malignant tumour progression and chemoresistance. As a well-known major driver of EMT, Snail expression has been reported to be correlated with cancer metastasis and poorer survival^29^. Our findings indicated the critical role of FBP1 in Snail-induced EMT and cancer progression in HCC, which is similar to that in breast cancer^9^ and gastric cancer^30^. Furthermore, GSEA indicated several enriched pathways deserving to be explored to explain the underlying mechanism of dysregulated FBP1 suppression. For example, the ERK and WNT pathways have been demonstrated to be involved in FBP1 regulation in cancers^10,12^. However, much effort should be made to validate these findings from GSEA.

Several questions remain to be further investigated. First, it is a limitation that we lack biochemical evidence showing that our ectopic FBP1 was enzymatically active. Whether FBP1 enzymatic activity or any other enzyme-activity-independent mechanism plays critical role in Snail-induced EMT remained to be clarified. Second, we found that FBP1 did not affect the expression of Snail, raising the question of how FBP1 suppressed the transcriptional activity of Snail. Two different mechanisms might help explain the phenomenon. One possibility is that FBP1 participates in a Snail subcellular location mechanism, such as nuclear transport, and then modulates Snail stability^31^. Another possibility is that FBP1 directly inhibits the Snail functional domain in the nucleus, similar to HIF in renal cancer^8^. Clarifying the subcellular location of FBP1 in Snail-induced EMT is critical to understanding the detailed interaction of Snail and FBP1. Last but not least, we found no significant correlation between FBP1 and Snail mRNA in HCC patients. The results must be explained with great caution. The difference between in vivo and in vitro experiments might be due to the underlying baseline characteristics (HCC aetiology, patient ethnicity, related-related characteristics) and need to be further validated.

## Conclusions

In summary, our study demonstrated that FBP1 was suppressed in Snail-induced EMT and HCC patients. Ectopic FBP1 expression hindered EMT and tumour growth induced by Snail overexpression. Several pathways, such as ESC, CSR and PDGF, could be further explored to explain the underlying mechanisms of FBP1 dysregulation. Loss of FBP1 indicated poorer prognosis and was an independent prognostic factor of HCC. Our study revealed an important role for FBP1 in Snail-induced EMT and prognostic prediction in HCC.

## Electronic supplementary material

Supplemental Table 1

Supplemental Table 2

Supplemental Table 3

Supplemental Table 4

Supplemental Figure 1

Supplemental Figure 2

Supplementary figure legends
