# Supplementary material for: Restoration of FBP1 suppressed Snail-induced epithelial to mesenchymal transition in hepatocellular carcinoma
Source: Cell Death Dis. 2018 Nov 14;9(11):1132. doi: 10.1038/s41419-018-1165-x (PMC6235921; doi:10.1038/s41419-018-1165-x)
Supplement: Supplementary file 1 — Supplemental Table 1 [file 41419_2018_1165_MOESM1_ESM.docx]

| **Supplemental Table 1 Overlapping gene set enrichment analyses terms between high Snail and low FBP1 group in 361 TCGA HCC** | | | | | | | | |
| --- | --- | --- | --- | --- | --- | --- | --- | --- |
|  | **High Snail** | | | | **Low FBP1** | | | |
| **Gene ontology (GO)** | | | | | | | | |
| **Term** | **SIZE** | **ES** | **NES** | **NOM p-val** | **SIZE** | **ES** | **NES** | **NOM p-val** |
| **C2 KEGG (3 terms)** | | | | | | | | |
| KEGG_NOTCH_SIGNALING_PATHWAY | 44 | 0.583 | 1.750 | 0.002 | 44 | -0.532 | -1.610 | 0.008 |
| KEGG_PATHOGENIC_ESCHERICHIA_COLI_INFECTION | 55 | 0.550 | 1.736 | 0.002 | 55 | -0.538 | -1.638 | 0.012 |
| KEGG_WNT_SIGNALING_PATHWAY | 144 | 0.443 | 1.548 | 0.026 | 144 | -0.471 | -1.642 | 0.008 |
| **C5** | | | | | | | | |
| **Biological process (90 terms)** | | | | | | | | |
| GO_CELL_PROJECTION_ASSEMBLY | 239 | 0.470 | 1.528 | 0.027 | 239 | -0.445 | -1.479 | 0.043 |
| GO_MYELOID_CELL_DIFFERENTIATION | 180 | 0.559 | 1.982 | <0.001 | 180 | -0.427 | -1.521 | 0.032 |
| GO_POSITIVE_REGULATION_OF_CYTOPLASMIC_TRANSPORT | 264 | 0.428 | 1.609 | <0.001 | 264 | -0.402 | -1.497 | 0.014 |
| GO_WNT_SIGNALING_PATHWAY | 329 | 0.378 | 1.435 | 0.034 | 329 | -0.432 | -1.587 | 0.002 |
| GO_REGULATION_OF_MULTI_ORGANISM_PROCESS | 441 | 0.405 | 1.630 | 0.008 | 441 | -0.390 | -1.498 | 0.022 |
| GO_MORPHOGENESIS_OF_AN_ENDOTHELIUM | 16 | 0.586 | 1.599 | 0.032 | 16 | -0.559 | -1.543 | 0.042 |
| GO_REGULATION_OF_MUSCLE_CELL_DIFFERENTIATION | 144 | 0.508 | 1.822 | <0.001 | 144 | -0.422 | -1.543 | 0.004 |
| GO_SMALL_GTPASE_MEDIATED_SIGNAL_TRANSDUCTION | 338 | 0.514 | 1.802 | <0.001 | 338 | -0.433 | -1.517 | 0.034 |
| GO_REGULATION_OF_EXTRACELLULAR_MATRIX_ORGANIZATION | 24 | 0.523 | 1.559 | 0.028 | 24 | -0.527 | -1.653 | 0.012 |
| GO_REGULATION_OF_ERYTHROCYTE_DIFFERENTIATION | 35 | 0.550 | 1.649 | 0.008 | 35 | -0.521 | -1.562 | 0.022 |
| GO_POSITIVE_REGULATION_OF_PROTEOLYSIS | 344 | 0.376 | 1.496 | 0.025 | 344 | -0.394 | -1.524 | 0.016 |
| GO_STRESS_ACTIVATED_PROTEIN_KINASE_SIGNALING_CASCADE | 97 | 0.481 | 1.671 | 0.004 | 97 | -0.431 | -1.484 | 0.038 |
| GO_NEGATIVE_REGULATION_OF_PEPTIDYL_SERINE_PHOSPHORYLATION | 21 | 0.604 | 1.658 | 0.008 | 21 | -0.536 | -1.528 | 0.046 |
| GO_REGULATION_OF_PROTEIN_COMPLEX_ASSEMBLY | 342 | 0.459 | 1.709 | <0.001 | 342 | -0.418 | -1.512 | 0.022 |
| GO_MYELOID_CELL_DEVELOPMENT | 39 | 0.557 | 1.644 | 0.006 | 39 | -0.600 | -1.772 | 0.002 |
| GO_REGULATION_OF_PHOSPHATASE_ACTIVITY | 117 | 0.446 | 1.565 | 0.012 | 117 | -0.429 | -1.513 | 0.016 |
| GO_ENDOMEMBRANE_SYSTEM_ORGANIZATION | 445 | 0.411 | 1.487 | 0.024 | 445 | -0.414 | -1.489 | 0.031 |
| GO_REGULATION_OF_STRIATED_MUSCLE_CELL_DIFFERENTIATION | 82 | 0.502 | 1.812 | <0.001 | 82 | -0.464 | -1.638 | 0.004 |
| GO_POSITIVE_REGULATION_OF_INTRACELLULAR_PROTEIN_TRANSPORT | 231 | 0.409 | 1.569 | 0.002 | 231 | -0.420 | -1.557 | <0.001 |
| GO_PROTEIN_DEPHOSPHORYLATION | 182 | 0.413 | 1.464 | 0.048 | 182 | -0.451 | -1.586 | 0.008 |
| GO_REGULATION_OF_LAMELLIPODIUM_ORGANIZATION | 33 | 0.568 | 1.748 | 0.002 | 33 | -0.519 | -1.498 | 0.045 |
| GO_REGULATION_OF_RESPONSE_TO_BIOTIC_STIMULUS | 225 | 0.395 | 1.527 | 0.025 | 225 | -0.402 | -1.485 | 0.016 |
| GO_REGULATION_OF_ACTIN_NUCLEATION | 25 | 0.625 | 1.738 | 0.002 | 25 | -0.560 | -1.480 | 0.038 |
| GO_SIGNAL_TRANSDUCTION_BY_PROTEIN_PHOSPHORYLATION | 390 | 0.440 | 1.698 | <0.001 | 390 | -0.386 | -1.454 | 0.027 |
| GO_REGULATION_OF_PHOSPHOPROTEIN_PHOSPHATASE_ACTIVITY | 56 | 0.458 | 1.469 | 0.045 | 56 | -0.503 | -1.603 | 0.002 |
| GO_MODULATION_BY_SYMBIONT_OF_HOST_CELLULAR_PROCESS | 26 | 0.540 | 1.600 | 0.018 | 26 | -0.597 | -1.662 | <0.001 |
| GO_POSITIVE_REGULATION_OF_INTRACELLULAR_TRANSPORT | 339 | 0.426 | 1.624 | <0.001 | 339 | -0.403 | -1.503 | 0.006 |
| GO_POSITIVE_REGULATION_OF_ERYTHROCYTE_DIFFERENTIATION | 22 | 0.614 | 1.714 | <0.001 | 22 | -0.526 | -1.510 | 0.027 |
| GO_MODIFICATION_BY_SYMBIONT_OF_HOST_MORPHOLOGY_OR_PHYSIOLOGY | 43 | 0.461 | 1.549 | 0.026 | 43 | -0.449 | -1.494 | 0.040 |
| GO_FC_RECEPTOR_SIGNALING_PATHWAY | 182 | 0.446 | 1.536 | 0.047 | 182 | -0.523 | -1.674 | 0.008 |
| GO_REGULATION_OF_WNT_SIGNALING_PATHWAY | 293 | 0.389 | 1.486 | 0.015 | 293 | -0.435 | -1.613 | 0.002 |
| GO_BONE_CELL_DEVELOPMENT | 20 | 0.670 | 1.728 | <0.001 | 20 | -0.619 | -1.568 | 0.021 |
| GO_CELLULAR_RESPONSE_TO_REACTIVE_OXYGEN_SPECIES | 100 | 0.505 | 1.878 | <0.001 | 100 | -0.389 | -1.484 | 0.023 |
| GO_NEURAL_TUBE_FORMATION | 91 | 0.481 | 1.549 | 0.027 | 91 | -0.470 | -1.460 | 0.049 |
| GO_EPHRIN_RECEPTOR_SIGNALING_PATHWAY | 84 | 0.546 | 1.788 | <0.001 | 84 | -0.520 | -1.629 | <0.001 |
| GO_ESTABLISHMENT_OF_CELL_POLARITY | 81 | 0.558 | 1.771 | 0.004 | 81 | -0.484 | -1.530 | 0.028 |
| GO_POSITIVE_REGULATION_OF_CELLULAR_PROTEIN_LOCALIZATION | 329 | 0.399 | 1.516 | 0.013 | 329 | -0.437 | -1.598 | 0.002 |
| GO_REGULATION_OF_CARDIAC_MUSCLE_CELL_DIFFERENTIATION | 19 | 0.618 | 1.770 | 0.002 | 19 | -0.536 | -1.515 | 0.035 |
| GO_THYMUS_DEVELOPMENT | 45 | 0.478 | 1.570 | 0.013 | 45 | -0.475 | -1.448 | 0.037 |
| GO_IN_UTERO_EMBRYONIC_DEVELOPMENT | 299 | 0.458 | 1.689 | 0.002 | 299 | -0.411 | -1.478 | 0.026 |
| GO_NEGATIVE_REGULATION_OF_DNA_BIOSYNTHETIC_PROCESS | 31 | 0.493 | 1.501 | 0.020 | 31 | -0.521 | -1.613 | 0.014 |
| GO_REGULATION_OF_INTRINSIC_APOPTOTIC_SIGNALING_PATHWAY | 130 | 0.384 | 1.474 | 0.025 | 130 | -0.446 | -1.630 | <0.001 |
| GO_REGULATION_OF_MYOTUBE_DIFFERENTIATION | 54 | 0.479 | 1.591 | 0.004 | 54 | -0.485 | -1.572 | 0.006 |
| GO_NEGATIVE_REGULATION_OF_HOMEOSTATIC_PROCESS | 117 | 0.495 | 1.704 | 0.006 | 117 | -0.439 | -1.486 | 0.028 |
| GO_POSITIVE_REGULATION_OF_EPITHELIAL_CELL_APOPTOTIC_PROCESS | 22 | 0.614 | 1.762 | <0.001 | 22 | -0.500 | -1.510 | 0.049 |
| GO_POSITIVE_REGULATION_OF_PROTEIN_LOCALIZATION_TO_NUCLEUS | 125 | 0.485 | 1.729 | 0.002 | 125 | -0.472 | -1.608 | 0.006 |
| GO_RESPONSE_TO_GAMMA_RADIATION | 49 | 0.517 | 1.645 | 0.004 | 49 | -0.501 | -1.507 | 0.038 |
| GO_REGULATION_OF_ADHERENS_JUNCTION_ORGANIZATION | 47 | 0.638 | 1.876 | <0.001 | 47 | -0.483 | -1.467 | 0.047 |
| GO_POSITIVE_REGULATION_OF_RESPONSE_TO_CYTOKINE_STIMULUS | 30 | 0.490 | 1.478 | 0.047 | 30 | -0.556 | -1.630 | 0.020 |
| GO_REGULATION_OF_INTRACELLULAR_PROTEIN_TRANSPORT | 347 | 0.384 | 1.478 | 0.012 | 347 | -0.395 | -1.476 | 0.016 |
| GO_REGULATION_OF_EMBRYONIC_DEVELOPMENT | 105 | 0.495 | 1.745 | 0.002 | 105 | -0.435 | -1.487 | 0.032 |
| GO_REGULATION_OF_CANONICAL_WNT_SIGNALING_PATHWAY | 226 | 0.378 | 1.465 | 0.025 | 226 | -0.431 | -1.603 | <0.001 |
| GO_POSITIVE_REGULATION_OF_PROTEIN_POLYMERIZATION | 80 | 0.588 | 2.005 | <0.001 | 80 | -0.451 | -1.495 | 0.039 |
| GO_CELLULAR_SENESCENCE | 33 | 0.496 | 1.574 | 0.015 | 33 | -0.512 | -1.552 | 0.027 |
| GO_CELL_AGING | 66 | 0.448 | 1.579 | 0.011 | 66 | -0.473 | -1.615 | 0.006 |
| GO_POSITIVE_REGULATION_OF_ACTIN_FILAMENT_POLYMERIZATION | 61 | 0.608 | 2.037 | <0.001 | 61 | -0.476 | -1.531 | 0.037 |
| GO_NEGATIVE_REGULATION_OF_DEPHOSPHORYLATION | 68 | 0.435 | 1.469 | 0.050 | 68 | -0.431 | -1.464 | 0.037 |
| GO_CELLULAR_RESPONSE_TO_OXIDATIVE_STRESS | 175 | 0.443 | 1.727 | <0.001 | 175 | -0.372 | -1.481 | 0.016 |
| GO_REGULATION_OF_DEFENSE_RESPONSE_TO_VIRUS | 183 | 0.404 | 1.562 | 0.017 | 183 | -0.402 | -1.464 | 0.018 |
| GO_REGULATION_OF_NUCLEOCYTOPLASMIC_TRANSPORT | 210 | 0.453 | 1.631 | 0.006 | 210 | -0.415 | -1.457 | 0.038 |
| GO_NEGATIVE_REGULATION_OF_INTRINSIC_APOPTOTIC_SIGNALING_PATHWAY | 79 | 0.392 | 1.417 | 0.043 | 79 | -0.451 | -1.601 | 0.002 |
| GO_RAS_PROTEIN_SIGNAL_TRANSDUCTION | 137 | 0.522 | 1.738 | 0.002 | 137 | -0.500 | -1.656 | 0.006 |
| GO_POSITIVE_REGULATION_OF_NEURON_DEATH | 64 | 0.454 | 1.562 | 0.004 | 64 | -0.447 | -1.515 | 0.021 |
| GO_POSITIVE_REGULATION_OF_CELLULAR_COMPONENT_BIOGENESIS | 368 | 0.514 | 1.974 | <0.001 | 368 | -0.393 | -1.437 | 0.042 |
| GO_REGULATION_OF_CYTOPLASMIC_TRANSPORT | 450 | 0.410 | 1.559 | <0.001 | 450 | -0.394 | -1.486 | 0.017 |
| GO_HIPPO_SIGNALING | 26 | 0.608 | 1.489 | 0.048 | 26 | -0.606 | -1.496 | 0.043 |
| GO_NEGATIVE_REGULATION_OF_ERBB_SIGNALING_PATHWAY | 43 | 0.479 | 1.491 | 0.046 | 43 | -0.566 | -1.704 | 0.008 |
| GO_REGULATION_OF_RESPONSE_TO_CYTOKINE_STIMULUS | 133 | 0.457 | 1.636 | 0.004 | 133 | -0.446 | -1.541 | 0.033 |
| GO_REGULATION_OF_CYTOSKELETON_ORGANIZATION | 462 | 0.509 | 1.832 | <0.001 | 462 | -0.432 | -1.503 | 0.039 |
| GO_NEGATIVE_REGULATION_OF_TRANSFERASE_ACTIVITY | 336 | 0.348 | 1.402 | 0.029 | 336 | -0.397 | -1.561 | 0.008 |
| GO_ERYTHROCYTE_HOMEOSTASIS | 69 | 0.489 | 1.661 | 0.006 | 69 | -0.428 | -1.486 | 0.026 |
| GO_POSITIVE_REGULATION_OF_MULTI_ORGANISM_PROCESS | 145 | 0.409 | 1.515 | 0.015 | 145 | -0.456 | -1.602 | 0.002 |
| GO_WNT_SIGNALING_PATHWAY_CALCIUM_MODULATING_PATHWAY | 35 | 0.538 | 1.589 | 0.021 | 35 | -0.521 | -1.553 | 0.021 |
| GO_REGULATION_OF_FIBROBLAST_PROLIFERATION | 79 | 0.504 | 1.765 | <0.001 | 79 | -0.452 | -1.495 | 0.036 |
| GO_REGULATION_OF_DEFENSE_RESPONSE_TO_VIRUS_BY_HOST | 130 | 0.361 | 1.403 | 0.041 | 130 | -0.384 | -1.451 | 0.018 |
| GO_POSITIVE_REGULATION_OF_MUSCLE_CELL_DIFFERENTIATION | 82 | 0.507 | 1.779 | <0.001 | 82 | -0.405 | -1.451 | 0.036 |
| GO_NEGATIVE_REGULATION_OF_APOPTOTIC_SIGNALING_PATHWAY | 186 | 0.388 | 1.557 | 0.007 | 186 | -0.389 | -1.485 | 0.012 |
| GO_RETROGRADE_TRANSPORT_ENDOSOME_TO_GOLGI | 63 | 0.501 | 1.533 | 0.036 | 63 | -0.510 | -1.496 | 0.027 |
| GO_APOPTOTIC_SIGNALING_PATHWAY | 276 | 0.403 | 1.579 | 0.004 | 276 | -0.388 | -1.479 | 0.029 |
| GO_POSITIVE_REGULATION_OF_ERBB_SIGNALING_PATHWAY | 34 | 0.556 | 1.662 | 0.006 | 34 | -0.503 | -1.586 | 0.014 |
| GO_POSITIVE_REGULATION_OF_APOPTOTIC_SIGNALING_PATHWAY | 162 | 0.412 | 1.545 | 0.017 | 162 | -0.457 | -1.584 | 0.010 |
| GO_REGULATION_OF_DEPHOSPHORYLATION | 144 | 0.451 | 1.609 | 0.004 | 144 | -0.460 | -1.608 | 0.002 |
| GO_POSITIVE_REGULATION_OF_PROTEIN_COMPLEX_ASSEMBLY | 175 | 0.489 | 1.797 | <0.001 | 175 | -0.460 | -1.624 | 0.002 |
| GO_POSITIVE_REGULATION_OF_INTRINSIC_APOPTOTIC_SIGNALING_PATHWAY | 47 | 0.476 | 1.545 | 0.018 | 47 | -0.506 | -1.587 | 0.012 |
| GO_REGULATION_OF_APOPTOTIC_SIGNALING_PATHWAY | 342 | 0.392 | 1.604 | 0.004 | 342 | -0.406 | -1.549 | 0.010 |
| GO_GLIAL_CELL_DEVELOPMENT | 72 | 0.466 | 1.622 | 0.004 | 72 | -0.424 | -1.470 | 0.034 |
| GO_ACTIN_NUCLEATION | 21 | 0.563 | 1.626 | 0.031 | 21 | -0.572 | -1.672 | 0.014 |
| GO_NEGATIVE_REGULATION_OF_CYSTEINE_TYPE_ENDOPEPTIDASE_ACTIVITY | 84 | 0.460 | 1.715 | 0.002 | 84 | -0.402 | -1.443 | 0.028 |
| GO_REGULATION_OF_ERBB_SIGNALING_PATHWAY | 78 | 0.538 | 1.737 | <0.001 | 78 | -0.515 | -1.659 | 0.006 |
| GO_POSITIVE_REGULATION_OF_BINDING | 120 | 0.433 | 1.615 | 0.002 | 120 | -0.401 | -1.456 | 0.022 |
| **Cellular component (7 terms)** | | | | | | | | |
| GO_CYTOPLASMIC_REGION | 270 | 0.480 | 1.783 | <0.001 | 270 | -0.390 | -1.442 | 0.035 |
| GO_ORGANELLE_SUBCOMPARTMENT | 295 | 0.397 | 1.456 | 0.035 | 295 | -0.435 | -1.528 | 0.010 |
| GO_ANCHORING_JUNCTION | 463 | 0.559 | 2.034 | <0.001 | 463 | -0.444 | -1.594 | 0.017 |
| GO_TRANS_GOLGI_NETWORK | 183 | 0.428 | 1.491 | 0.039 | 183 | -0.469 | -1.574 | 0.014 |
| GO_LATE_ENDOSOME | 199 | 0.406 | 1.546 | 0.012 | 199 | -0.385 | -1.475 | 0.044 |
| GO_ENDOSOMAL_PART | 400 | 0.396 | 1.487 | 0.038 | 400 | -0.404 | -1.478 | 0.049 |
| GO_CELL_SUBSTRATE_JUNCTION | 377 | 0.557 | 1.989 | <0.001 | 377 | -0.490 | -1.722 | 0.004 |
| **Molecular function (25 terms)** | | | | | | | | |
| GO_TRANSCRIPTION_COREPRESSOR_ACTIVITY | 207 | 0.401 | 1.498 | 0.026 | 207 | -0.439 | -1.616 | 0.004 |
| GO_PHOSPHATASE_REGULATOR_ACTIVITY | 83 | 0.449 | 1.578 | 0.008 | 83 | -0.436 | -1.513 | 0.028 |
| GO_PROTEIN_PHOSPHATASE_BINDING | 116 | 0.463 | 1.619 | 0.004 | 116 | -0.483 | -1.687 | 0.004 |
| GO_HORMONE_RECEPTOR_BINDING | 161 | 0.379 | 1.468 | 0.033 | 161 | -0.425 | -1.582 | 0.004 |
| GO_PROTEIN_TYROSINE_PHOSPHATASE_ACTIVITY | 99 | 0.453 | 1.577 | 0.019 | 99 | -0.435 | -1.488 | 0.043 |
| GO_PROTEIN_COMPLEX_SCAFFOLD | 63 | 0.474 | 1.555 | 0.031 | 63 | -0.479 | -1.585 | 0.014 |
| GO_PROTEIN_KINASE_C_BINDING | 48 | 0.508 | 1.754 | 0.002 | 48 | -0.486 | -1.619 | 0.002 |
| GO_TRANSCRIPTION_FACTOR_ACTIVITY_RNA_POLYMERASE_II_TRANSCRIPTION_FACTOR_BINDING | 128 | 0.446 | 1.594 | 0.010 | 128 | -0.445 | -1.494 | 0.021 |
| GO_ACTIN_FILAMENT_BINDING | 112 | 0.550 | 1.884 | <0.001 | 112 | -0.431 | -1.494 | 0.036 |
| GO_GTPASE_BINDING | 281 | 0.436 | 1.543 | 0.025 | 281 | -0.461 | -1.613 | 0.014 |
| GO_PHOSPHATASE_BINDING | 157 | 0.442 | 1.568 | 0.008 | 157 | -0.463 | -1.648 | 0.006 |
| GO_NUCLEOSIDE_TRIPHOSPHATASE_REGULATOR_ACTIVITY | 313 | 0.502 | 1.736 | 0.002 | 313 | -0.433 | -1.555 | 0.029 |
| GO_RNA_POLYMERASE_II_DISTAL_ENHANCER_SEQUENCE_SPECIFIC_DNA_BINDING | 62 | 0.485 | 1.592 | 0.019 | 62 | -0.495 | -1.521 | 0.038 |
| GO_GTPASE_ACTIVITY | 232 | 0.445 | 1.642 | <0.001 | 232 | -0.399 | -1.495 | 0.031 |
| GO_KINASE_REGULATOR_ACTIVITY | 178 | 0.467 | 1.762 | <0.001 | 178 | -0.372 | -1.399 | 0.036 |
| GO_ENHANCER_BINDING | 89 | 0.460 | 1.561 | 0.027 | 89 | -0.489 | -1.536 | 0.012 |
| GO_RHO_GTPASE_BINDING | 73 | 0.505 | 1.662 | 0.008 | 73 | -0.480 | -1.549 | 0.032 |
| GO_PHOSPHOLIPASE_BINDING | 17 | 0.632 | 1.660 | 0.006 | 17 | -0.535 | -1.472 | 0.046 |
| GO_PHOSPHOPROTEIN_BINDING | 59 | 0.527 | 1.769 | <0.001 | 59 | -0.511 | -1.650 | 0.015 |
| GO_CORE_PROMOTER_SEQUENCE_SPECIFIC_DNA_BINDING | 94 | 0.479 | 1.622 | 0.017 | 94 | -0.480 | -1.632 | 0.004 |
| GO_PROTEIN_PHOSPHORYLATED_AMINO_ACID_BINDING | 24 | 0.647 | 1.839 | 0.004 | 24 | -0.534 | -1.544 | 0.043 |
| GO_ENZYME_ACTIVATOR_ACTIVITY | 441 | 0.470 | 1.727 | <0.001 | 441 | -0.390 | -1.486 | 0.037 |
| GO_RNA_POLYMERASE_II_TRANSCRIPTION_COFACTOR_ACTIVITY | 87 | 0.432 | 1.489 | 0.041 | 87 | -0.489 | -1.562 | 0.010 |
| GO_RNA_POLYMERASE_II_CORE_PROMOTER_SEQUENCE_SPECIFIC_DNA_BINDING | 49 | 0.458 | 1.520 | 0.039 | 49 | -0.444 | -1.467 | 0.038 |
| GO_ACTIVATING_TRANSCRIPTION_FACTOR_BINDING | 56 | 0.469 | 1.508 | 0.042 | 56 | -0.516 | -1.609 | 0.012 |
| **C6 Oncological signature (4 terms)** | | | | | | | | |
| PDGF_ERK_DN.V1_DN | 139 | 0.479 | 1.671 | 0.002 | 139 | -0.447 | -1.624 | 0.002 |
| ESC_J1_UP_LATE.V1_DN | 174 | 0.441 | 1.760 | <0.001 | 174 | -0.371 | -1.460 | 0.013 |
| DCA_UP.V1_DN | 166 | 0.352 | 1.378 | 0.038 | 166 | -0.368 | -1.404 | 0.014 |
| CSR_EARLY_UP.V1_UP | 148 | 0.531 | 1.776 | 0.008 | 148 | -0.466 | -1.569 | 0.016 |
