# Supplementary material for: Restoration of FBP1 suppressed Snail-induced epithelial to mesenchymal transition in hepatocellular carcinoma
Source: Cell Death Dis. 2018 Nov 14;9(11):1132. doi: 10.1038/s41419-018-1165-x (PMC6235921; doi:10.1038/s41419-018-1165-x)
Supplement: Supplementary file 2 — Supplemental Table 2 [file 41419_2018_1165_MOESM2_ESM.docx]

| **Supplemental Table 2 Univariate analyses of factors associated with RFS and OS in our cohort** | | | | |
| --- | --- | --- | --- | --- |
| **Factors** | **RFS** | | **OS** | |
|  | **HR（95%CI）** | **P** | **HR（95%CI）** | **P** |
| Sex: male v female | 0.719（0.441-1.171） | 0.185 | 0.637（0.368-1.105） | 0.109 |
| Age: ≤50 v >50 years | 0.943（0.701-1.269） | 0.698 | 0.964（0.700-1.328） | 0.822 |
| ALT: ≥40 v <40 IU/L | 1.007（0.742-1.368） | 0.963 | 1.152（0.837-1.188） | 0.385 |
| Tbil: ≥17 v <17 μmol/L | 1.302（0.946-1.792） | 0.105 | 1.188（0.847-1.655） | 0.318 |
| ALB: ≥35 v < 35 g/L | 0.626（0.353-1.111） | 0.109 | 0.516（0.286-0.932） | **0.028** |
| AFP: ≥20 v <20 ng/ml | 1.334（0.937-1.899） | 0.110 | 1.239（0.845-1.816） | 0.272 |
| Cirrhosis: yes v no | 0.987（0.691-1.409） | 0.941 | 1.239（0.845-1.816） | 0.272 |
| Diameter:＞5 v ≤5 cm | 2.350（1.676-3.296） | **＜0.001** | 2.441（1.680-3.546） | **＜0.001** |
| Encapsulation: complete v incomplete or absence | 0.503（0.372-0.681） | **＜0.001** | 0.490（0.355-0.677） | **＜0.001** |
| Satellite nodules: yes v no | 2.121（1.559-2.886） | **＜0.001** | 2.394（1.724-3.324） | **＜0.001** |
| PVTT: yes v no | 3.064（2.133-4.402） | **＜0.001** | 3.033（2.093-4.395） | **＜0.001** |
| Edmonson grade: III+IV v I+II | 1.649（1.173-2.319） | **0.004** | 1.559（1.087-2.234） | **0.02** |
| TNM: II+III+IV v I | 2.647（1.956-3.581） | **＜0.001** | 3.079（2.222-4.268） | **＜0.001** |
| BCLC: B+C v A | 2.731（2.015-3.70） | **＜0.001** | 3.203（2.311-4.441） | **＜0.001** |
| FBP1 expression: high v low | 0.552 (0.409-0.747) | **＜0.001** | 0.511 (0.369-0.708) | **＜0.001** |
| **Abbreviations:** AFP, alpha-fetoprotein; ALT, alanine aminotransferase; ALB, albumin; BCLC stage, Barcelona Clinic Liver Cancer stage; FBP1, fructose-1,6-bisphosphatase 1; TBil, total bilirubin; TNM stage, tumor-node-metastasis stage; PVTT, portal vein tumor thrombosis | | | | |
