# Supplementary material for: Restoration of FBP1 suppressed Snail-induced epithelial to mesenchymal transition in hepatocellular carcinoma
Source: Cell Death Dis. 2018 Nov 14;9(11):1132. doi: 10.1038/s41419-018-1165-x (PMC6235921; doi:10.1038/s41419-018-1165-x)
Supplement: Supplementary file 3 — Supplemental Table 3 [file 41419_2018_1165_MOESM3_ESM.docx]

| **Supplemental Table 3 Included Patient characteristics in GS14520 cohort** | | | | |
| --- | --- | --- | --- | --- |
|  |  | FBP1^Low^  (n=107) | BP1^High^  (n=108) | P |
| Age（years） | ≤60 | 86 | 88 | 0.864 |
|  | ＞60 | 21 | 20 |  |
| Gender | male | 94 | 92 | 0.690 |
|  | female | 13 | 16 |  |
| ALT (U/L) | ≤50 | 64 | 64 | 0.934 |
|  | >50 | 43 | 44 |  |
| Main tumor size (cm) | ≤5 | 60 | 78 | **0.014** |
|  | >5 | 47 | 30 |  |
| Multinodular | no | 79 | 91 | 0.067 |
|  | yes | 28 | 17 |  |
| Cirrhosis | no | 6 | 12 | 0.218 |
|  | yes | 101 | 96 |  |
| TNM Stage | I | 36 | 53 | **0.022** |
|  | III | 71 | 55 |  |
| BCLC stage | 0-A | 73 | 91 | **0.006** |
|  | B+C | 34 | 17 |  |
| AFP (ng/ml) | ≤300 | 47 | 71 | **0.001** |
|  | >300 | 60 | 37 |  |
| **Abbreviations:** AFP, alpha-fetoprotein; ALT, alanine aminotransferase; BCLC stage, Barcelona Clinic Liver Cancer stage; FBP1, fructose-1,6-bisphosphatase 1; TNM stage, tumor-node-metastasis stage | | | | |
