# Supplementary material for: Restoration of FBP1 suppressed Snail-induced epithelial to mesenchymal transition in hepatocellular carcinoma
Source: Cell Death Dis. 2018 Nov 14;9(11):1132. doi: 10.1038/s41419-018-1165-x (PMC6235921; doi:10.1038/s41419-018-1165-x)
Supplement: Supplementary file 4 — Supplemental Table 4 [file 41419_2018_1165_MOESM4_ESM.doc]

| **Supplemental Table 4 Univariate and Multivariate Analyses for Overall Survival and Recurrence-free Survival in included GSE14520 Cohort** | | | | | | | | |
| --- | --- | --- | --- | --- | --- | --- | --- | --- |
|  | Overall Survival | | | | Recurrence-free Survival | | | |
| Factors | Univariate analysis | | Multivariate analysis | | Univariate analysis | | Multivariate analysis | |
|  | HR (95% CI) | P | HR (95% CI) | P | HR (95% CI) | P | HR (95% CI) | P |
| Age (≤60 vs ＞60) | 0.806 (0.454-1.432) | 0.462 |  |  | 0.963 (0.611-1.517) | 0.870 |  |  |
| Gender female vs male | 0.606 (0.293-1.257) | 0.178 |  |  | 0.470 (0.246-0.898) | **0.022** | - | - |
| ALT (>50 vs ≤50） | 1.085 (0.704-1.671) | 0.713 |  |  | 1.264 (0.882-1.812) | 0.202 |  |  |
| Main tumor size (cm) (>5 vs ≤5) | 2.068 (1.342-3.185) | **0.001** | - | - | 1.507 (1.041-2.180) | **0.030** | - | - |
| Multinodular (yes vs no) | 1.555 (0.962-2.514) | 0.072 |  |  | 1.150 (0.744-1.777) | 0.528 |  |  |
| Cirrhosis (yes vs no) | 4.738 (1.165-19.272) | **0.030** | 4.123 (1.011-16.821) | **0.048** | 2.256 (0.992-5.130) | 0.052 |  |  |
| TNM Stage (III vs I+II) | 2.821 (1.719-4.631) | **<0.001** | 1.786 (1.027-3.105) | **0.040** | 2.191 (1.485-3.234) | **<0.001** | 1.825 (1.208-2.756) | **0.004** |
| BCLC Stage (B+C vs 0+A) | 3.381 (2.166-5.276) | **<0.001** | 2.340 (1.427-3.838) | **0.001** | 1.447 (1.207-1.735) | **<0.001** | 1.295 (1.071-1.567) | **0.008** |
| AFP (ng/ml) (>300 vs ≤300) | 1.651 (1.075-2.537) | **0.022** | - | **-** | 1.273 (0.889-1.822) | 0.187 | - | **-** |
| FBP1 (High vs Low) | 0.460 (0.294-0.718) | **0.001** | 0.588 (0.373-0.929) | **0.023** | 0.623 (0.434-0.895) | **0.010** | - | **-** |
| **Abbreviations:** AFP, alpha-fetoprotein; ALT, alanine aminotransferase; BCLC stage, Barcelona Clinic Liver Cancer stage; FBP1, fructose-1,6-bisphosphatase 1; TNM stage, tumor-node-metastasis stage | | | | | | | | |
