# Supplementary figures and images for: Restoration of FBP1 suppressed Snail-induced epithelial to mesenchymal transition in hepatocellular carcinoma
Source: Cell Death Dis. 2018 Nov 14;9(11):1132. doi: 10.1038/s41419-018-1165-x (PMC6235921; doi:10.1038/s41419-018-1165-x)

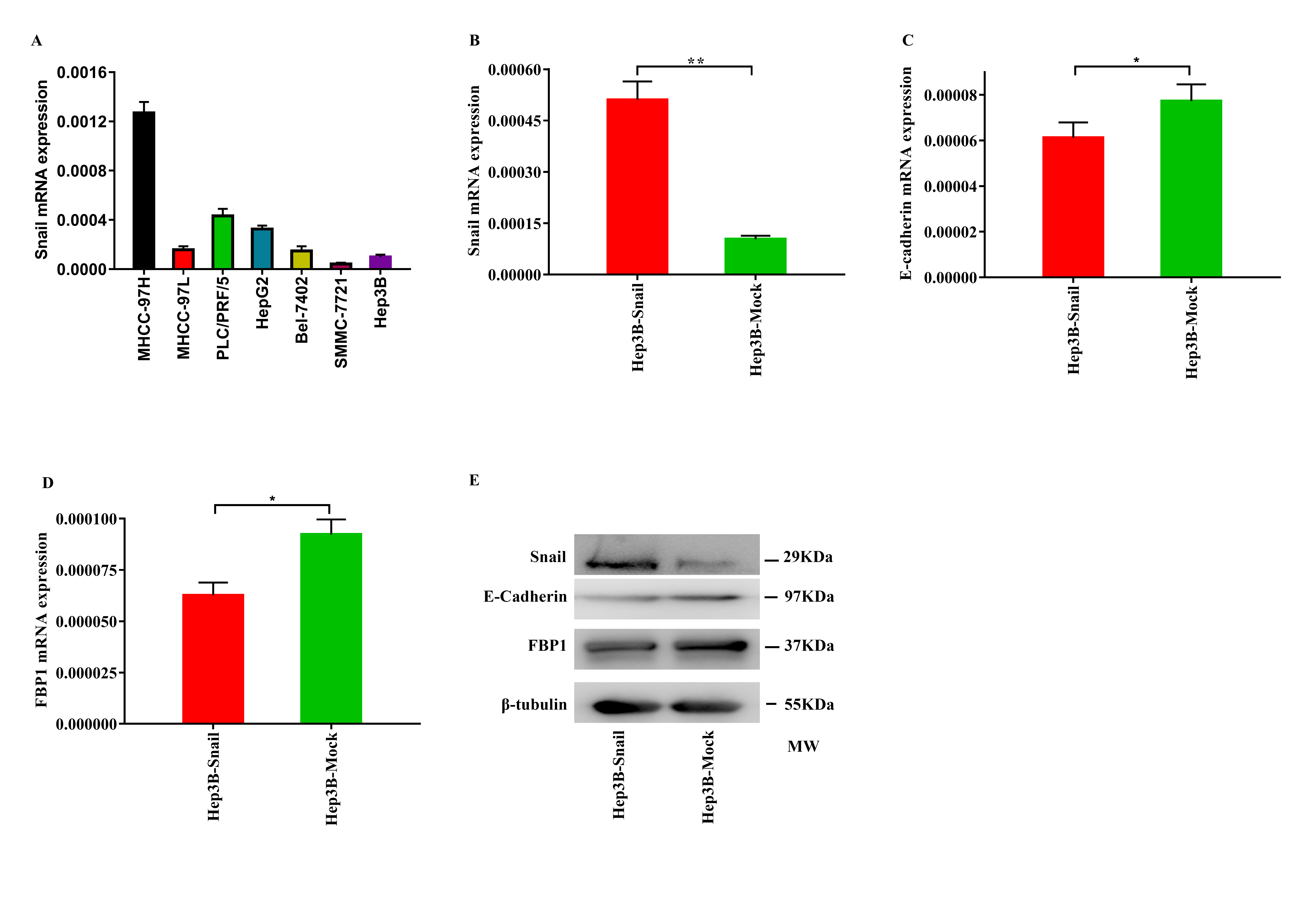

Supplement: Supplementary file 5 — Supplemental Figure 1 [file 41419_2018_1165_MOESM5_ESM.tif]

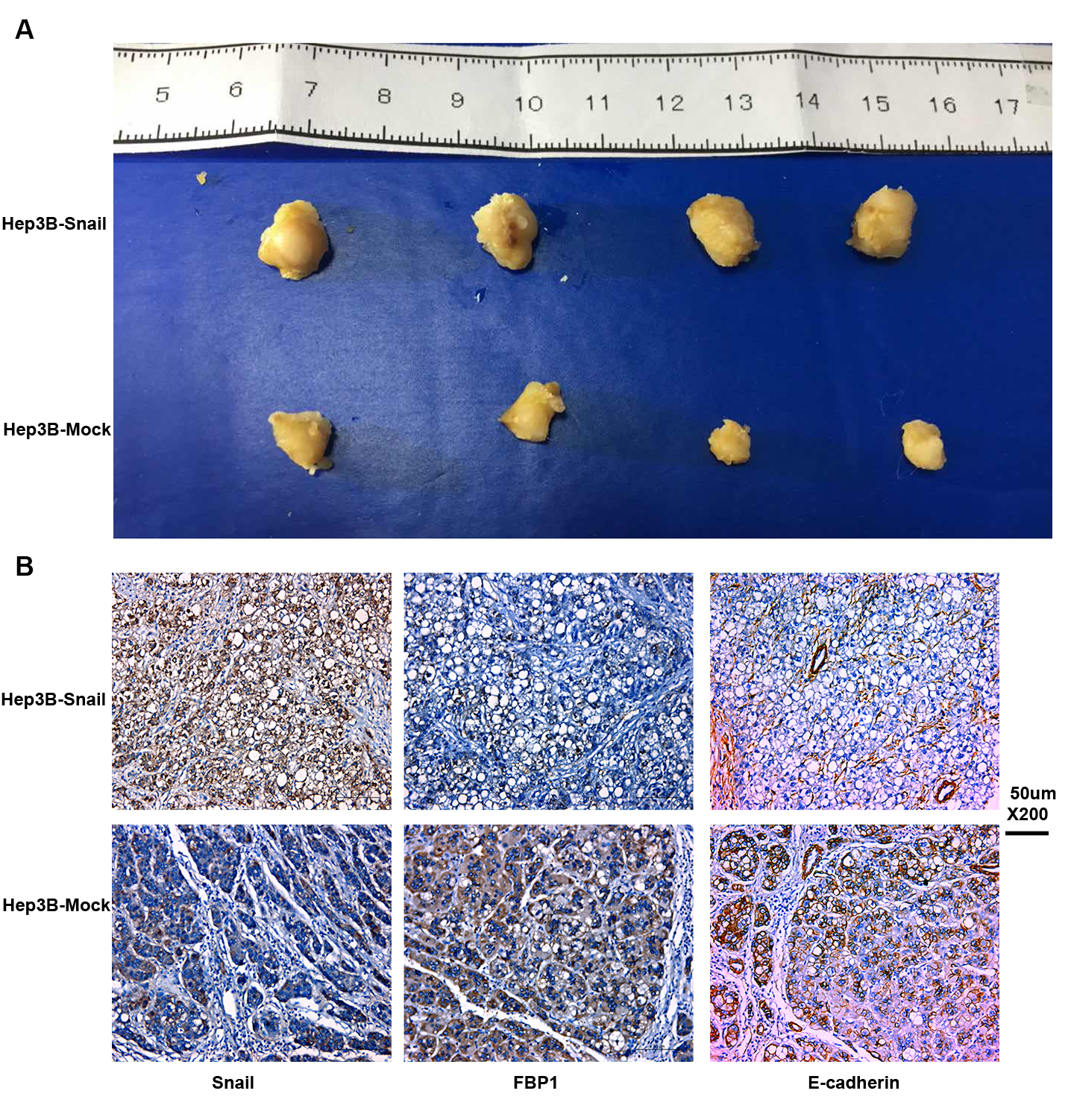

Supplement: Supplementary file 6 — Supplemental Figure 2 [file 41419_2018_1165_MOESM6_ESM.tif]
