# Supplementary material for: Restoration of FBP1 suppressed Snail-induced epithelial to mesenchymal transition in hepatocellular carcinoma
Source: Cell Death Dis. 2018 Nov 14;9(11):1132. doi: 10.1038/s41419-018-1165-x (PMC6235921; doi:10.1038/s41419-018-1165-x)
Supplement: Supplementary file 7 — Supplementary figure legends [file 41419_2018_1165_MOESM7_ESM.docx]

**Figure legends for supplemental figures**

**Supplemental Figure 1** Expression of FBP1 was suppressed during Snail-induced EMT.

(A) The expression of Snail in HCC cell lines using tubulin for normalization. Snail level was highest in MHCC-97H but lowest in SMMC-7721. (B) Quantitative real-time PCR analysis showed significantly higher expression of Snail in Hep-3B-Snail than Hep-3B-Mock cells. (C) Quantitative real-time PCR analysis showed significantly lower expression of E-cadherin in Hep-3B-Snail than Hep-3B-Mock cells. (D) Quantitative real-time PCR analysis showed significantly lower expression of FBP1 in Hep-3B-Snail than Hep-3B-Mock cells. (H) Western blot analysis showed significantly lower expression of E-cadherin and FBP1 following Snail over-expression. * P<0.05, ** P<0.01, compared to control. MW: molecular weight. All data are based on three independent repeats.

**Supplemental Figure 2** Snail induced tumour growth and suppressed E-cadherin and FBP1 expression in Hep-3B cells.

(A) Representative images of subcutaneous tumour in nude mice induced by Hep-3B-Snail and Hep-3B mock cells. (B) The representative images of Snail, FBP1 and E-cadherin expression in HCC tissues of transplanted tumour. The magnifications used were 200(X). The number of mice in each group in **A** is 4.
